# Screening Marine Natural Products for New Drug Leads against Trypanosomatids and Malaria

**DOI:** 10.3390/md18040187

**Published:** 2020-03-31

**Authors:** María Álvarez-Bardón, Yolanda Pérez-Pertejo, César Ordóñez, Daniel Sepúlveda-Crespo, Nestor M. Carballeira, Babu L. Tekwani, Sankaranarayanan Murugesan, Maria Martinez-Valladares, Carlos García-Estrada, Rosa M. Reguera, Rafael Balaña-Fouce

**Affiliations:** 1 Department of Biomedical Sciences; University of León, 24071 León, Spain; malvb@unileon.es (M.Á.-B.); myperp@unileon.es (Y.P.-P.); c.ordonez@unileon.es (C.O.); dsepc@unileon.es (D.S.-C.); rmregt@unileon.es (R.M.R.); 2 Department of Chemistry, University of Puerto Rico, Río Piedras 00925-2537, San Juan, Puerto Rico; nestor.carballeira1@upr.edu; 3 Department of Infectious Diseases, Division of Drug Discovery, Southern Research, Birmingham, AL 35205, USA; btekwani@southernresearch.org; 4 Department of Pharmacy, Birla Institute of Technology and Science, Pilani Campus, Vidya Vihar, Pilani 333031, India; murugesan@pilani.bits-pilani.ac.in; 5 Department of Animal Health, Instituto de Ganadería de Montaña (CSIC-Universidad de León), Grulleros, 24346 León, Spain; mmarva@unileon.es; 6 INBIOTEC (Instituto de Biotecnología de León), Avda. Real 1-Parque Científico de León, 24006 León, Spain; carlos.garcia@inbiotec.com

**Keywords:** neglected tropical diseases, trypanosomatids, malaria, high-throughput screening, phenotypic screening, target-based screening, marine pharmacology, chloroquine derivatives

## Abstract

Neglected Tropical Diseases (NTD) represent a serious threat to humans, especially for those living in poor or developing countries. Almost one-sixth of the world population is at risk of suffering from these diseases and many thousands die because of NTDs, to which we should add the sanitary, labor and social issues that hinder the economic development of these countries. Protozoan-borne diseases are responsible for more than one million deaths every year. Visceral leishmaniasis, Chagas disease or sleeping sickness are among the most lethal NTDs. Despite not being considered an NTD by the World Health Organization (WHO), malaria must be added to this sinister group. Malaria, caused by the apicomplexan parasite *Plasmodium falciparum*, is responsible for thousands of deaths each year. The treatment of this disease has been losing effectiveness year after year. Many of the medicines currently in use are obsolete due to their gradual loss of efficacy, their intrinsic toxicity and the emergence of drug resistance or a lack of adherence to treatment. Therefore, there is an urgent and global need for new drugs. Despite this, the scant interest shown by most of the stakeholders involved in the pharmaceutical industry makes our present therapeutic arsenal scarce, and until recently, the search for new drugs has not been seriously addressed. The sources of new drugs for these and other pathologies include natural products, synthetic molecules or repurposing drugs. The most frequent sources of natural products are microorganisms, e.g., bacteria, fungi, yeasts, algae and plants, which are able to synthesize many drugs that are currently in use (e.g. antimicrobials, antitumor, immunosuppressants, etc.). The marine environment is another well-established source of bioactive natural products, with recent applications against parasites, bacteria and other pathogens which affect humans and animals. Drug discovery techniques have rapidly advanced since the beginning of the millennium. The combination of novel techniques that include the genetic modification of pathogens, bioimaging and robotics has given rise to the standardization of High-Performance Screening platforms in the discovery of drugs. These advancements have accelerated the discovery of new chemical entities with antiparasitic effects. This review presents critical updates regarding the use of High-Throughput Screening (HTS) in the discovery of drugs for NTDs transmitted by protozoa, including malaria, and its application in the discovery of new drugs of marine origin.

## 1. Introduction

The marine environment, by being so diverse, is a unique place to find novel biomaterials. In this world, there is severe competition for survival, as well as environmental pressure. This unique, but reachable biodiversity utilizes unique metabolites for either defense, attack or signaling. These metabolites, many without counterparts in the terrestrial world, continue to hold potential for new human applications, mostly in the field of pharmaceuticals and novel pharmacophores [1]. Some marine organisms from which bioactive metabolites can be isolated include sponges, tunicates, corals and mollusks. In addition, microorganisms associated with marine organisms such as the actinomycetes, fungi, dinoflagellates, cyanobacteria and even noncultivable symbionts are also promising sources of interesting bioactive metabolites [2]. Amongst the several marine-derived compounds already on the market, we find drugs to treat cancer, viral infections, neuropathic pain and even hypertriglyceridemia [3]. Marine-derived anticancer products include Adcetris^®^, Halaven^®^, Yondelis®, and Cytosar-u^®^ [4]. Adcetris^®^ (Brentuximab vedotin) is an antibody-drug conjugate active against CD30-positive cancer cells such as those associated with classical Hodgkin lymphoma [5]. The key component of Adcetris® is a peptide, originally isolated from the marine mollusk *Dolabella auricularia*, related to the dolastatins, which displays high antitumor activity [6]. Halichondrin B, isolated from the sponge *Halichondria okadai*, inspired the development of Halaven^®^ (Eribulin mesylate) [7], marketed by Eisai in Japan and also approved by the US Food and Drug Administration (FDA). It is recommended for the treatment of patients with breast cancer and liposarcoma by inhibiting the microtubule assembly [8]. Yondelis^®^ (Trabectedin), a drug developed by PharmaMar SA based on Ecteinascidin 743, a marine natural product isolated from the tunicate *Ecteinascidia turbinata*, has been approved for use in Europe, Russia and South Korea for the treatment of advanced soft-tissue sarcoma [9]. The US-FDA has approved this drug to treat two types of soft tissue sarcomas that metastasize to different parts of the body and cannot be treated by standard surgery [10]. Cytosar-u^®^ (cytarabine) was developed to treat different forms of leukemia, including myeloid and meningeal leukemia. The original natural product from which cytarabine was modeled is spongothymidine, which was isolated from the sponge *Cryptotheca crypta* [4,11]. However, despite these promising drugs, no marine-based drugs have been developed for NTDs and malaria. This review provides critical updates on High-Throughput Screening (HTS) techniques for NTDs transmitted by major trypanosomatids and malaria, and their possible application to the discovery of new drugs from marine sources.

## 2. Trypanosomatids-Borne NTDs and Malaria

Parasitic protozoa of the family trypanosomatidae are responsible for a number of deadly NTDs, mostly in low-income communities of Africa, Asia and South America [12]. The prevalence and incidence of these diseases can be alleviated through the implementation of appropriate public health and hygiene measures, but the pharmacological approach is the only useful tool that can control them when an epidemic breaks out. However, the figures published by the WHO for NTDs and malaria are alarming; one sixth of the global population is affected by at least one of these diseases, especially in some areas of Africa and Asia [13,14,15]. NTDs and malaria are deadly when left untreated, and their impact should not only be measured in terms of epidemiological data, but also in terms of the devastation produced in families, whose incomes can be seriously compromised on account of illness, lost work days due to disability or the high cost of treatment [16].

### 2.1. Human African Trypanosomiasis

Human African Trypanosomiasis (HAT) is a zoonosis caused by *T. brucei* and transmitted by the tsetse fly in central and sub-Saharan African countries [17,18,19]. The prevalence of this disease is showing a clear decrease, partly due to the control efforts made over the past 20 years. Statistics for the last 10 years (period 2008–2016) revealed that the number of HAT cases (both gambiense and rodhesiense) reported and registered was ca. 55,000, with the incidence in 2018 being just 977 [20]. These figures are the lowest since systematic statistics of this disease were established 80 years ago, and clearly improve the estimations made in the WHO roadmap in 2012 [21]. However, 57 million people still remain at risk of contracting the disease in 36 countries, (the Democratic Republic of the Congo is home to about 70% of this population). The estimated Disability Adjusted Life Year (DALY) for HAT in 2010 was 560,000, which represents a 72% decrease in comparison to the 1990 statistics [22].

The severity of HAT depends on the subspecies of the parasite involved in the infection. *T. brucei* gambiense causes chronic infection and is responsible for 98% of cases. The disease may go undetected for months or years, before neurological symptoms appear in the advanced stages [23]. However, *T. brucei rhodesiense* causes acute infection in only 2% of cases [24]. Both forms of the disease can be fatal when left untreated; however, self-healing has been described in patients affected by the gambiense form [25].

### 2.2. American Trypanosomiasis or Chagas Disease

Chagas disease is also a zoonotic disease caused by *T. cruzi* and transmitted by the feces of triatomine kissing bugs [26,27,28]. Chagas disease is limited to the South American subcontinent, but is an emerging disease in USA [29] and Europe [30]—mainly in the southern countries—due to migratory flows over the past 20 years. More than 8 million people (down from 30 million in 1990) suffer from this disease worldwide and nearly 10,000 people die each year from complications related to the disease. Annual DALYs due to American trypanosomiasis are estimated to be 236,100, which represents only a 3% reduction over the period 2005–2015 [22]. The disease is curable if treatment begins within the early stages of the infection. However, the chronic phase of the disease, developed by 30% of infected persons, is responsible for cardiac [31] and digestive [32] disorders that can be fatal, even when treated. Although the transmission vector is absent from the European continent, Chagas disease is an emerging disease in southern European countries due to infected migrants from endemic areas of Latin America [33]. Contact with infected blood during blood transfusions [34] or organ transplants [35] and vertical transmission from pregnant women are common ways of acquiring the disease in both endemic and nonendemic areas [36].

### 2.3. Leishmaniasis

Leishmaniasis is a complex of diseases produced by parasites of the genus *Leishmania* and transmitted by phlebotomine sand flies. Leishmaniasis includes at least three forms of disease presentation and one relapse form [37,38,39]. The cutaneous form of the disease is mainly produced by *L. major* and *L. tropica* in the Old World and by *L. braziliensis* in the New World. It is the less severe form of the disease, but is responsible for sores and scars that can be disfiguring [37,38,39,40]. The mucocutaneous presentation, caused by the *L. amazonensis* complex in the New World, is a more severe and stigmatizing form of the disease. The infection usually progresses from a simple sore at the bite site to the complete destruction of the mucous membranes of the mouth and nose [37,38,39,41]. Finally, visceral leishmaniasis is the most severe presentation of the disease. It is produced by *L. donovani* and *L. infantum* in the Old World and by *L. chagasi* (*infantum*) in the New World [37,38,39,42]. Swelling of the liver and spleen, together with renal dysfunction, may lead to death without treatment. Visceral leishmaniasis may evolve into a postkala azar dermal leishmaniasis (PKDL), a rare skin form of the disease that occurs after the failure of classical antimony therapy [43]. The current incidence of the disease is 50,000 to 90,000 new cases of visceral leishmaniasis and 700,000 to 1 million of the different forms of cutaneous leishmaniasis, with a total of 26,000 to 65,000 deaths due exclusively to the visceral presentation [44,45]. The estimated DALYs in 2015 were almost 1.4 million, with a 38% increase in 2005–2015, mainly (>99%) due to visceral leishmaniasis [22]. Several eradication campaigns have been carried out in the Indian subcontinent, reducing the incidence of the disease in India, Nepal and Bangladesh by more than 50% [46]. However, the emergence of resistance to first-line drugs because of inappropriate application or undue treatment interruption has created resistant strains of the parasites [47].

### 2.4. Malaria or Paludism

Malaria is a major health problem in developing countries. Malaria occurs due to infection by a species of apicomplexan endoparasites of the genus *Plasmodium*, which are transmitted by *Anopheles* sp. mosquitoes [15]. According to the latest WHO report, approximately 3.4 billion people were living in malaria risk areas in 2012, with approximately 70% of malaria cases coming from 11 endemic countries, i.e., 10 in Africa and India. WHO estimated that 219 million people were affected worldwide, with the infamous figure of 435,000 malaria deaths in 2017 [48]. Between 2000 and 2017, the worldwide annual incidence of malaria declined by 36%, and the annual death rate declined by 60% [49]. However, the continuous decrease in the number of malaria cases in the Indian subcontinent contrasts with the high incidence in endemic African countries [50]. DALYs lost due to malaria were close to 56 million in 2015, which represents a 38.3% reduction since 2005. *P. falciparum* was responsible for 99% of malaria cases in the African continent and in Asia, while *P. vivax* accounts for approximately 9% of malaria cases worldwide, with the latter being the dominant species outside Africa. Like other diseases, children under the age of 5 are the most vulnerable population group and represented 61% of malaria deaths in 2017 [48].

## 3. Prevention and Lack of Vaccines Against NTDs

One of the most discouraging facts for the prevention of these diseases is the lack of effective vaccines that can prevent outbreaks in the human population. Most of these diseases induce immunity and, therefore, the challenge with live, attenuated or part of the parasites is an acceptable hypothesis to prevent future infections. In the 1980s, during the Iran–Iraq war, a massive preventive immunization campaign against cutaneous leishmaniasis (leishmanization) was carried out, in which Iran vaccinated its army with an attenuated strain of *L. major* with good results and few side effects. [51]. Some vaccines have been licensed to prevent *L. infantum*-canine leishmaniasis [52]. However, an effective and safe vaccine against visceral leishmaniasis in humans is still not available. Several prototype vaccines have been tested in human communities with only moderate or medium results. The first malaria vaccine that reached clinical phase III was RTS,S (Mosquirix™), which was developed by GlaxoSmithKline (GSK) and Walter Reed Army Institute of Research (WRAIR) (USA) from the circumsporozoite antigen obtained from attenuated *P. falciparum* sporozoites. The results obtained with more than 15,000 participants younger than 17 months of age (6,500 from 6 to 12 weeks of age) from seven sub-Saharan countries between 2009 and 2014 were modest (around 40% efficacy), but improved the health conditions and economic development of the regions where the program was implemented [53]. In spite of these results, the WHO recommended implementation studies with this vaccine in a 4-dose schedule for children 5–17 months old in several endemic African countries [54].

## 4. The Pharmacological Approach

**Figure 1:**
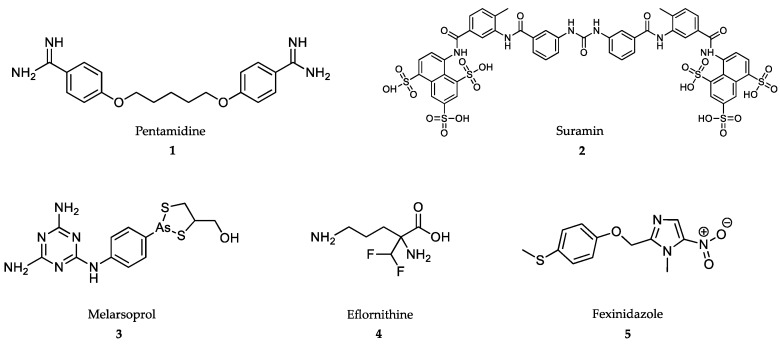
Drugs in clinical use against Human African Trypanosomiasis (HAT).

**Figure 2:**
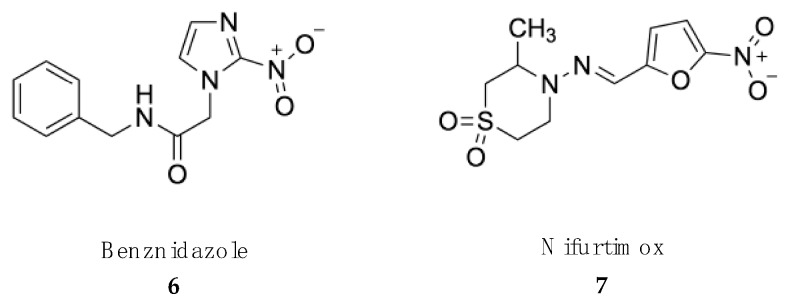
Drugs in clinical use against American trypanosomiasis (Chagas disease).

**Figure 3:**
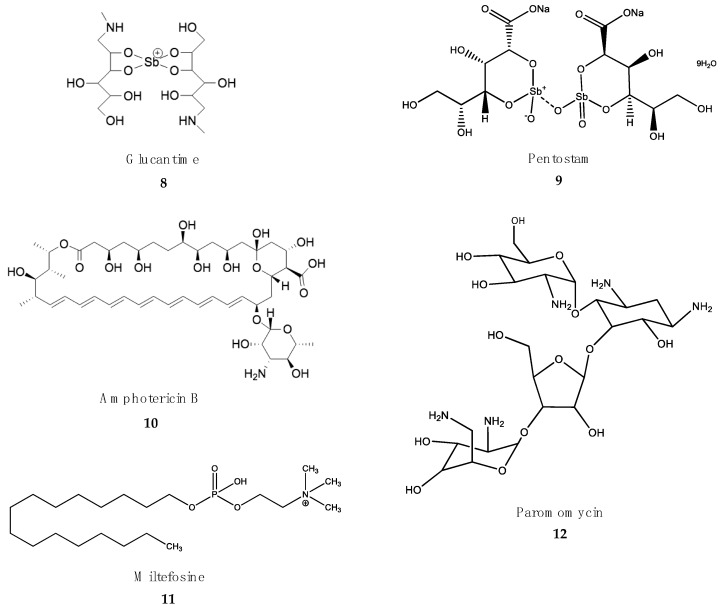
Drugs in clinical use against leishmaniasis.

**Figure 4:**
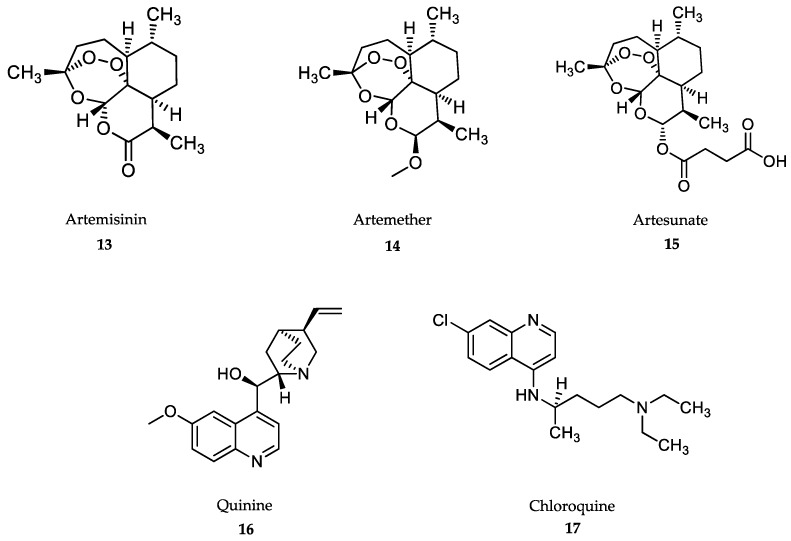
Drugs in clinical use against malaria.

Most of the drugs used against NTDs are outdated and associated with adverse side effects and/or treatment failures and relapses due to drug resistance [55,56]. However, the importance of these medicines to save lives is such that most of them are included in the WHO Essential Medicines List (Figure 1, Figure 2, Figure 3 and Figure 4).

### 4.1. Treatment of African Trypanosomiasis

At present, there are four drugs globally approved for the treatment of HAT: pentamidine, melarsoprol, eflornithine and nifurtimox, the latter only in combination with eflornithine (NECT) [57]. Fexinidazole, the first all-oral treatment for sleeping sickness, has been recently approved in Democratic Republic of Congo for the treatment of HAT (vide infra) (Figure 1).

Pentamidine **(1)** is an aromatic diamidine synthesized in the 1950s that is prescribed for the early nonneurological stage of sleeping sickness caused by *T. brucei gambiense* in West Africa [58], but not for intermediate or late stages [59]. The cationic nature of terminal amidine groups enables the interaction of pentamidine with anionic groups of DNA at the minor groove, thus inhibiting DNA synthesis [60]. In addition, pentamidine inhibits S-adenosylmethionine decarboxylase, and as a consequence, causes growth arrest [61]. Due to its cationic nature, pentamidine uses membrane cell transporters, such as AqP2 aquaglyceroporine, for its internalization. Mutations in this transporter are at the origin of the mixed arsenical-pentamidine coresistances observed in the field [62]. Pentamidine has low oral bioavailability and should be administered by intramuscular injection at 4 mg/kg body weight at 24 h intervals for one week [57,63] (Table 1). Pentamidine is generally well tolerated, but it may cause pain at the site of injection, vomiting, hypotension, tachycardia and skin irritation [58]. 

Suramin **(2)** is a polysulfonated napthylamine-based drug that is effective against early stages of rhodesiensis HAT [64,65]. Suramin is poorly absorbed by the oral route and has to be intravenously administered at a dose of 5 mg/kg body weight on day one, followed by 10 mg/kg body weight on day 3 and 20 mg/kg body weight on days 5, 11, 17, 23 and 30 [57]. The strong binding of suramin to plasmatic proteins provides this molecule with an estimated half-life of 44–54 days [66], and justifies the low penetration into the central nervous system cerebrospinal fluid [67]. Suramin enters into the parasite compartment through receptor-mediated endocytosis [68] and disturbs the trypanosomal enzymes of glycosomes, thus preventing the formation of procyclic forms [69]. Major adverse side-effects for suramin include hypersensitivity, nephropathy, peripheral neuropathy and bone marrow toxicity [70]. 

Melarsoprol **(3)** is an arsenite-based drug used for the treatment of second-stage of gambiense sleeping sickness when the patient develops neurological symptoms [57]. The drug is highly effective when administered in a 10-day intravenous infusion program consisting of 2.2 mg/kg body weight/day (more than 93.9% cure rate) [57,71]. Melarsoprol is a prodrug that has to be metabolized to an active molecule of As^3+^ (melarsen-oxide), the metabolite found in CNS [72]. As^3+^ is the only active species able to inhibit the pathways for scavenging free radicals in trypanosomatids [73]. Another mechanism of action of melarsoprol is the inhibition of the adenosine (P2 carrier) [74,75] and aquaporin (AqP2) transporters of the parasite [76]. Mutations at the AqP2 locus are responsible for resistance to melarsoprol and also for cross-resistance to pentamidine [77]. Arsenical encephalopathy is one of the late-syndromes found after chronic treatment of HAT with melarsoprol and other As-based drugs [78].

Eflornithine **(4)** or difluoromethylornithine (DFMO) is a fluorinated analogue of ornithine that was repurposed early from its use against cancer. Eflornithine is an irreversible inhibitor of ornithine decarboxylase (ODC), the key enzyme of putrescine biosynthesis. This drug was originally used against the late-CNS-stage of gambiense HAT, but not against rodhesiense HAT [79]. The seminal work by Bacchi’s group (Pace University, New York, USA) showed the curative effect of the drug on murine in vivo infections with *T. brucei brucei* by irreversible inhibition of parasite ODC [79,80,81]. 

Eflornithine is the most advanced target-based drug used against NTDs, but its pharmacokinetic limitations reduce its trypanostatic/cydal potential. Firstly, DFMO is poorly bioavailable and requires intravenous administration. Secondly, bulky doses of 100 to 150 (children) mg/kg body weight every 6 h up to a total of 56 doses administered during 14 days are necessary to obtain a 99% cure rate against gambiense HAT, and may lead to noncompliance [57]. The long durations of drug treatments are necessary due to its short blood half-life (estimated to be 1.5 to 5 h). Another reason is the low rate of drug penetration through the blood-brain barrier to kill the parasites [82,83,84,85]. Adverse drug-effects include diarrhea, dizziness, headaches and seizures. More severe symptoms include anemia, leukopenia and thrombocytopenia [86].

The most advanced therapy against gambiense HAT is NECT, a combination of eflornithine with the trypanocidal nitroheterocyclic-class drug nifurtimox [87,88]. NECT cuts the treatment duration to almost 50% with only 14 total infusions. In this case, nifurtimox should be administered orally in three daily doses for 10 days. In a multicenter randomized study conducted in the Democratic Republic of the Congo, NECT achieved a more than 97% cure rate [89].

Fexinidazole **(5)**, a 5-nitroimidazole derivative, is a DNA synthesis inhibitor developed by Sanofi in collaboration with the Drugs for Neglected Diseases initiative (DNDi), for the oral treatment of HAT [90]. Fexinidazole is the first oral-drug treatment for both the early and late stages of the disease [91,92].

### 4.2. Treatment of American Trypanosomiasis

The two drugs currently used against both the acute and chronic phases of American trypanosomiasis, namely, benznidazole and nifurtimox, belong to the large group of nitroheterocyclic compounds [93,94] (Figure 2).

Benznidazole **(6)** is a pro-drug with nitroimidazole structure, which was originally developed for the treatment of HAT. The recommended dose of benznidazole is 5–7 mg/kg body weight orally, divided into 2–3 daily doses for 60 days in adults and 5–10 mg/kg body weight orally, divided into 2–3 daily doses for 60 days for children up to 12 years old [95]. The mechanism of action of nitroheterocyclic compounds is not clear. Activation by a specific type I (oxygen sensitive) nitroreductase (NTR-1), which is absent in the host cells, generates a series of reactive intermediates responsible for the trypanocydal effect, which may also be the cause of their mutagenic risk to the host [96,97]. Benznidazole benefits are higher during acute stages of the disease in adults and for children and young adults with chronic intermediate Chagas disease [98]. This is explained by its full oral bioavailability, with plasma half-lives ranging from 11 to 13 h in adults [99]. Since the half live is shorter in children, dose adjustment is required [100]. Congenital transmission of Chagas disease is one of the most important challenges in both endemic and nonendemic countries. Benznidazole prevents congenital transmission when administered to women of childbearing age, which can be an important strategy to prevent the disease in newborns [101]. Benznidazole tolerance is satisfactory, since no serious side effects have been described in treated patients. The side effects include allergy, dermopathy, nausea and vomiting. Less frequent are polyneuropathy and bone marrow depression [102].

Nifurtimox **(7)** is a nitrofurazone developed as second-line option for the treatment of American trypanosomiasis [103]. Several clinical studies have shown that nifurtimox administered to adults at 8–10 mg/kg body weight orally in 3 daily doses for 90 days or at 15–20 mg/kg body weight orally or as 4 doses divided daily for 90 days to children achieved a cure rate of 80–90% [104]. Similar to other nitroheterocycles, reduction of the nitro group by specific NTR-2 promotes the accumulation of nitrogen free radicals that cause cell death [105]. Nifurtimox is extensively metabolized after oral administration with an elimination half-life ranging from 2.0 to 5.4 h [106]. Adverse side effects to nifurtimox are frequent and include anorexia, vomiting, gastric pain, insomnia, headache, myalgia and convulsions [107].

Despite their long therapeutic use, nitroheterocycle-based compounds have inspired new drugs entering clinical trials against NTDs. The 5-nitroimidazole fexinidazole has strong trypanocidal activity and is in phase III clinical development for HAT, supported by the DNDi [92,108]. For its part, DNDI-0690, a substituted nitroimidazooxazine based on the structure of the antitubercular (S)-PA-824, has been selected to enter phase I trials in the treatment of visceral leishmaniasis [109].

### 4.3. Treatment of Leishmaniasis

Since leishmaniasis is a complex of several diseases, different treatments are currently in use. These include different drugs, administration guidelines or combinations of drugs, depending on the geographical area and the presentation of the disease [110]. Five drugs are being used as monotherapy or in combination (Figure 3). However, despite the good results shown over the years, serious problems of resistance, lack of efficacy and toxicity recommend the introduction of new drugs [111,112].

Pentavalent antimonials. Similar to HAT, the first approaches to fighting leishmaniasis during the colonial era were based on organometallic derivatives of As and Sb [113]. Meglumine antimoniate (Glucantime) **(8)** and sodium stibogluconate (Pentostam) **(9)** are Sb^V^-based drugs that are being used as monotherapy or in combination with amphotericin B (AmpB) or miltefosine [114]. Sb^V^ derivatives are actually prodrugs that must be reduced by parasite reductases to the active Sb^III^ species [115]. Both Glucantime and Pentostam are poorly absorbed orally, and therefore, intramuscular or subcutaneous administration is recommended. In the Indian subcontinent and Africa, Pentostam is administered at a regime of 20–30 mg/kg body weight/day for 25–30 days [116]. The mechanism of action of pentavalent antimonials is not fully known. The active Sb^III^ species inhibit several enzymes of energy metabolism, cause the oxidation of fatty acids and induce thiol redox imbalance in leishmania amastigotes [113]. The excessive use of these drugs has resulted in the emergence of resistant *Leishmania* sp. strains. In addition, high levels of arsenic in drinking water in some regions of the Indian subcontinent may be responsible for natural resistance to these drugs [117]. The interruption of Sb^V^ treatment can cause relapses and the development of PKDL [118]. Antimony-based medicines are prescribed to youth over 15 years and adults up to 40 years of age. Cardiotoxicity increases in people below and above these ages. Arthralgia, myalgia, hepatotoxicity, pancreatitis and nephrotoxicity are other common toxic adverse effects of antimony-based drugs [119].

Amphotericin B (AmpB) **(10)** is a polyene macrolide antifungal produced by streptomycetes. Several formulations of AmpB are marketed for antifungal and antiparasitic purposes. The deoxycholate salt (Fungizone^®^) is currently being replaced by lipid formulations, such as AmBisome^®^, which increases the bioavailability of the drug, reduces the dose and decreases the nephrotoxic adverse effects [120]. The mechanism of action of AmpB is based on the binding to specific sterols (ergosterol) of the external cell membrane of fungi and parasites, which produces watery pores that cause the loss of the electrochemical gradient and cell death [121].

The poor oral bioavailability of AmpB formulations require their administration by slow intravenous infusion. In India, intravenous infusion of deoxycholate amphotericin at 1 mg/kg body weight/day is almost 100% effective [116]. However, this treatment is tedious, requires hospitalization, and there is a risk of noncompliance [122]. However, the administration of a single injection of AmBisome^®^ at 10 mg/kg body weight produces a remarkable 96% cure rate, and is becoming the gold standard in India, where resistance to Sb^V^ is frequent [123]. A serious problem of AmpB-based drugs is their poor stability in the extreme climatic conditions of the endemic countries of East Africa [124]. In addition, full AmBisome^®^ treatment is unacceptably expensive for the target endemic countries. The registration and marketing of new lipid formulations of AmpB by local companies has lowered the price of the drug in India. AmpB is not completely safe, and adverse effects such as nephrotoxicity, hypokalemia and myocarditis are common [125].

Miltefosine. The alkylphospholipid miltefosine **(11)** was originally developed as an antitumoral, but quickly showed its utility as antileishmanial drug. The remarkable water-solubility of miltefosine due to its zwiterionic nature permits oral administration with high plasma bioavailability [126]. Several metabolic processes, including sterol and fatty acid synthesis and, eventually, the induction of programmed cell death of the parasite, are the proposed mechanisms of action of miltefosine [127]. In addition to these effects on the parasite, miltefosine is also an immunomodulator that promotes IL-12-dependent T helper response in the host, leading to parasite clearance [128]. Treatment with mitefosine at a dose of 50-100 mg/kg body weight/day for a total of 28 days [129,130] yields a cure rate of almost 95%. In addition to its good oral bioavailability, a second advantage of miltefosine is its long half-life (more than 1 week), that results into a 5- to 10-fold increase in plasma levels in steady-state conditions [129]. However, allometric administration in children is needed to adjust its efficacy [131]. Miltefosine is a safe medicine. Common adverse effects are intestinal cramps, vomiting, diarrhea and anorexia. Miltefosine is not recommended during pregnancy due to teratogenic issues [132].

Paromomycin **(12)** is an aminoglycoside antibiotic produced by streptomycetes that is effective against visceral leishmaniasis when combined with miltefosine. Intramuscular injection of paromomycin at 11 mg/kg body weight/day for 21 days as monotherapy has been shown to reduce parasite burden in visceral leishmaniasis cases in India [133], but not in East Africa, where it has to be administered along with antimony-based drugs or miltefosine. The painful intramuscular injections, nephrotoxicity and ototoxicity are amongst the common adverse effects described for this drug [134]. Pentamidine **(1)** is still in use in human immunodeficiency virus (HIV)-visceral leishmaniasis co-infections in some African countries, and in cases of cutaneous and mucocutaneous leishmaniasis in South America [135].

Several combinations of AmBisome + paromomycin and AmBisome + miltefosine, tested in Asia [136] and Pentostam + paromomycin in East Africa, reduce time of treatment and improve the compliance of the patients [137].

### 4.4. Treatment of Malaria

The treatment of malaria is determined by both the etiology (species of the genus *Plasmodium* responsible for the disease) and severity of the disease (complicated or uncomplicated). Preventive treatments for travelers visiting endemic countries are also important factors in the selection of the type of treatment of the disease [15,138].

Artemisinin-based drugs **(13-15)** (Figure 4) are a family of compounds derived from natural products isolated from the plant *Artemisia annua*. These compounds were identified and characterized within Research Project 523, developed in China during the Cultural Revolution, with the aim of eradicating malaria using traditional Chinese medicine [139]. Artemisinins are sesquiterpene lactones that provides the scaffold for the development of semisynthetic derivatives, such as artemether **(14)** and artesunate **(15)**, which are currently in use against both complicated and uncomplicated forms of falciparum malaria [15]. Both artemether and artesunate are prodrugs, which are transformed into the active dihydroartemisinin form [140]. Artemisinin **(13)** contains a 1,2,4 trioxane ring, which is bioactivated by Fe^2+^, resulting in reactive oxygen radicals that destroy the intraerithrocytic schizonts [141,142]. Artesunate is the drug of choice against severe falciparum malaria. Intramuscular or intravenous injections of artesunate at 4 mg/kg body weight twice on the first day, followed by 3 days’ treatment with artemisinin-based combined therapies (ACT), have significantly reduced malaria fatalities in Asia and Africa compared to traditional quinine treatment [143].

Artemether, given by intramuscular injection at 3.2 mg/kg body weight, followed by 1.6 mg/kg body weight daily, confers smaller benefits than artesunate, and is used as second-choice drug or when artesunate is not available [143]. For uncomplicated falciparum malaria, ACTs are recommended, namely, artemether-lumefantine or artesunate-mefloquine, amodiaquine or artesunate-mefloquine, amodiaquine or alternatively, sulfadoxime-pyrimethamine combinations [15,144]. In this case, the artemisinin derivative is given for three days within the fixed dose combination of the alternative drugs, preferably as oral tablets [15]. ACTs are rapidly and orally effective, with cure rates higher than 90% and with fairly affordable prices for endemic countries [145]. Artemisinins do not have serious adverse effects. Neurological and reproductive toxicity effects have been described only at higher nonclinical doses [146].

**Figure 5:**
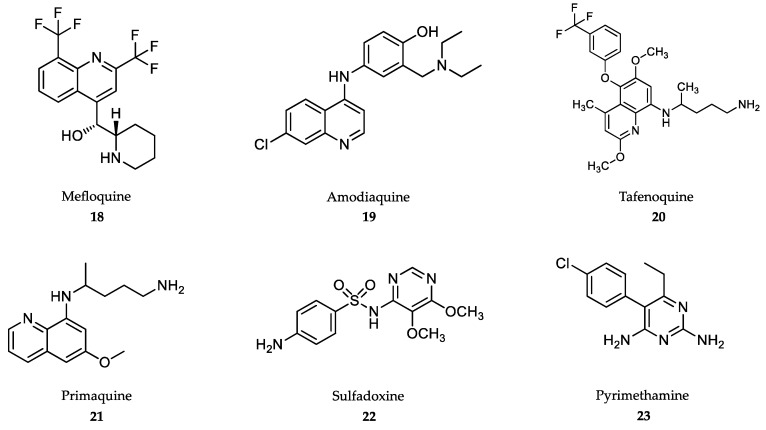
Drugs in clinical use against malaria.

Quinine and quinidine were the first compounds with a quinoline structure isolated from the bark of the cinchona tree in the early 19th century. These compounds were used as antimalarials until 2006. The antimalarial mechanism of action of quinoline-based compounds is attributed to the inhibition of hemozoin biocrystallization in the blood schizont stage of the parasite [147,148]. Quinine **(16)** and its semisynthetic derivative, chloroquine **(17)** (Figure 4), have been the most widely used antimalarial drugs to date, but the WHO has recommended that their use as a first-line antimalarial and as monotherapies should be discontinued as a result of increasing resistance rates since the 1980s [149]. However, the quinoline-scaffold has served as the inspiration for many others antimalarial drugs still in use, mostly in combination therapies against uncomplicated malaria presentations (Figure 5).

Mefloquine **(18)** is used in combination with artesunate for uncomplicated falciparum malaria, chloroquine resistance and also for the prevention of malaria in travelers [150]. Mefloquine is orally administered in tablets at a dose of 5-11 mg/kg body weight/day for 3 days along with artesunate for the blood stage of the disease [143]. Similar, to other quinolines, mefloquine also has very high bioavailability with a long half-life, i.e., from 2 to 5 weeks [151]. Common side effects in both adults and children include nausea, vomiting, diarrhea, headaches and cutaneous rash [152]. Neurological adverse effects are rare and include hallucinations, anxiety and depression, which prevent its use in patients with psychiatric disorders [153]. Next, 4-aminoquinoline, amodiaquine **(19)** is used in combination with artesunate for the treatment of uncomplicated malaria, but not as a preventive drug [154]. Similar to chloroquine, amodiaquine is fully metabolized by CYP450-2C8, but its de-ethylated metabolite still retains high antimalarial activity [155]. Amodiaquine is administered orally against *P. falciparum*-susceptible strains at 7.5-15 mg/kg body weight/day for 3 days along with artesunate [143]. Hepatitis and agranulocytosis were seen in patients taking amodiaquine for prophylaxis, which has led to its recommended discontinuation for this indication. Other adverse effects include headaches, trouble seeing, seizures and cardiac arrest. [152].

Antimalarials derived from the 8-aminoquinoline scaffold (Figure 5), tafenoquine **(20)**, and primaquine **(21)**, are recommended in combination with other antimalarials for the prevention of relapse of *P. vivax* and *P. ovale* infections, and by themselves as primary prophylaxis for travelers visiting endemic areas with high incidence of *P. vivax* [15,143,156]. Unlike 4-aminoquinoline antimalarials, 8-aminoquinolines kill the dormant hypnozoite liver stage, which is responsible for relapses, even when the blood stages are fully cleared [157]. Tafenoquine, administered at a single oral dose of 300 mg [158] in combination with the schizonticide, has a considerable advantage over primaquine, which is used at a dose of 0.25 mg/kg body weight/day for a 14-day course [143,159]. These drugs are contraindicated in patients with genetic deficiency of glucose 6-phosphate dehydrogenase due to severe hemolytic anemia [160]. Unlike primaquine, tafenoquine has been linked to transient mild elevation of liver serum enzymes during therapy [152].

Sulfadoxine **(22)** and pyrimethamine **(23)** were introduced in combination therapy as antimalarials, and they are currently administered along with artesunate in the treatment of uncomplicated, chloroquine-resistant falciparum malaria [15,143,161]. The synergy between both drugs is due to the fact that both inhibit folic acid synthesis by competing with dihydropteroate synthetase, which is an enzyme necessary for the conversion of p-aminobenzoic acid to folic acid (sulfadoxine), and dihydrofolate reductase (pyrimethamine), thereby blocking the biosynthesis of purines and pyrimidines in the parasite [162]. The combination of both drugs is greatly synergistic, with good oral bioavailability and long half-lives. A single dose of 25 mg/kg body weight sulfadoxine + 1.25 mg/kg body weight pyrimethamine is administered, along with intramuscular injections of artesunate to cure sensitive forms of uncomplicated falciparum malaria [19,143]. Preventive administration of sulfadoxine/pyrimethamine combination is an effective therapy to reduce the cases of malaria during pregnancy in Africa, although supplementation with folic acid is recommended [163]. Adverse effects include diarrhea, rash, itching, headache and hair loss. Its use is not recommended for patients with liver or kidney diseases [152].

## 5. Current tools for Drug Screening

Over the past two decades, as a result of the initiative of international stakeholders led by nonprofit research and development organizations, the search for new drugs against NTDs has taken a giant step forward. DNDi, Medicines for Malaria Venture (MMV), NGOs, academic and institutional centers, and private-public-partnerships with pharmaceutical companies such as GSK, Tres Cantos, Madrid (Spain) or Novartis GNF, San Diego (USA) have opened their robotic facilities and huge libraries of compounds to external researchers. These partnerships have resulted in significant successes in the discovery of new drugs against NTDs and malaria.

The use of HTS strategies for the identification of biologically active natural products is less frequent compared to that of synthetic compounds. More than 60% of small molecules approved between 1981 and 2014 against cancer were developed from a natural product or one of its pharmacophores [164]. Only 1% of the papers published in this same period used HTS technology [165]. Finally, this proportion becomes almost nonexistent when we talk about natural products of marine origin.

As a starting point, a high level of structural diversity is necessary from both small molecules and biological extracts which can be obtained by sampling the diverse marine taxonomy or by harvesting them in unexploited ecological niches. To give a few examples, it is worth mentioning the effort made by public institutions such as the National Cancer Institute of the United States (NCI) USA under the Program for the Discovery of Natural Products Discovery (NPNPD), which owns one of the largest and most diverse collections of natural extracts and natural products in the world [166]. This library is freely available in 384-well plates and is HTS-eligible for the research community. On the other hand, Fundación Medina (Granada, Spain) has a library of more than 130,000 extracts and semipurified fractions, which is currently the largest chemical space of natural products obtained from a large sampling of diverse geographical locations [167]. From the side of private enterprises, Pharmamar (Madrid, Spain), which is responsible for the development and commercialization of the antitumor drug trabectedin, has the world’s largest collection of natural products from marine origin, with approximately 200,000 samples of macroorganisms and microorganisms.

Empirical and nonempirical target-based screenings are current drug discovery strategies to identify new antiparasitic hits for further preclinical and clinical studies. There are pros and cons that can determine the choice of the screening method when establishing a new drug discovery campaign against NTDs [168]. On the one hand, phenotypic screening assays are unbiased and more relevant than target-based screenings, since neither lead to prejudices in the selection of compounds related to their mechanism of action, nor are accessibility issues present for the compound to the specific target. However, with these results, it is difficult to obtain conclusions regarding quantitative structure-activity relationships (QSAR), and complicated deconvolution studies are required to determine the possible mechanisms of action of the selected lead compounds to improve their effectiveness in a rational way [169]. On the other hand, target-based screens are more rational when a robust target is available. It is easier to improve the new compounds based on computational 3D docking studies. However, the relevance of this approach is usually lower, because the compounds are usually modified by the host or pathogen cells, and they barely reach the target in the active form [170]. In conclusion, although both are valid paradigms, historically, target-based screening has been the method of choice to identify best-in-class drugs, whereas phenotypic screens have served to identify first-in-class drugs [171]. Once the most promising hits are identified, new simulation-based computational analyses [172] and mammalian cell toxicity tests [173] are performed with a threefold objective: (i) to avoid toxic compounds, (ii) to perform QSAR analysis in order to introduce chemical modifications that improve the pharmacokinetic and pharmacodynamic properties of the compounds, and (iii) to avoid compounds which require complex synthesis at the pilot or industrial plant scales.

### 5.1. Phenotypic vs. Target-Based Screening in Trypanosomatids

The low throughput of the previously employed whole cell-based phenotypic models, together with the revolution in genomics and 3D-assisted prediction models, have led to support for the "target-first-then-phenotypic" strategy in drug discovery for many years [171]. If a validated drug target and a good chemical scaffold to selectively interact with the target are available, a rational structure-based drug discovery approach may be adapted for the synthesis of thousands of new compounds in the search for drug candidates. In general terms, target-based drug screening is easier to implement and provides continuous feedback from which to introduce rational modifications in the molecule that improve the interactions with its target through in-silico 3D predictions [174]. In the case of trypanosomes and malaria, a pharmacological target must have a number of characteristics, namely, (i) the target should be essential for the survival of the pathogen; (ii) should be druggable; (iii) should be structurally different from the heterologous form occurring in the host; and (iv) should be differently expressed in the parasite with respect to the host [175].

ODC, the key enzyme of polyamine biosynthesis, is the only consolidated target in *T. brucei*, although it is not in *T. cruzi*, *Leishmania* parasites and *Plasmodium* spp. [176]. The irreversible inhibition of this enzyme, differences in the rate of turnover and structural differences with the host protein, and the differential expression of the ODC-encoding gene, make ODC the ideal target for the development eflornithine, a drug for the treatment of HAT [177]. Sterol 14α-demethylase, a CYP monooxygenase that catalyzes the removal of the 14α-methyl group from eburicol [178], is another example of selective target of drug discovery for Chagas disease [179]. Two triazole antifungals, posaconazole and E1224 (a ravuconazole pro-drug), were developed against this target and submitted to Chagas disease clinical trials. Posaconazole and E1224 showed a transient suppressive effect on parasite clearance, but both failed during the follow-up [180,181]. Similarly, cyclin-dependent Cdc2-related kinase 12 (CRK12) was proposed as a potential druggable target in kinetoplastids [182]. The pyrazolopyrimidine GSK-3186899 identified in target-based screening showed excellent in vitro and in vivo antileishmanial effect when administered orally to mice infected with *L. donovani*. The in vivo efficacy, novel mechanism of action and safety profile of GSK-3186899 supported the advancement of this compound for definitive phase I clinical trials [183]. N-myristoyltransferase, an enzyme responsible for posttranslational protein modification in fungi and protozoa but not in mammals, was identified as a robust target in *T. brucei* [184]. It was consolidated after HTS of a library with 62,000 compounds [185], which yielded the optimized lead DDD85646. This is a pyrazole sulfonamide with good results in experimental infections of *T. b. brucei* and *T. b. rodhesiense* in mice [186]. 

Other targets that were genetically validated in kinetoplastids are the enzymes of both folate and unconjugated pteridines [187]. These include pteridine reductase 1 (PTR1) and the bifunctional dihydrofolate reductase-thymidylate synthase (DHFR-TS) [188]. Both enzymes have good draggability and have been assayed under HTS technologies in *T. brucei* [189] and *L. major* [190]. Trypanothione is the key molecule used by trypanosomatids for modulating oxidative stress in place of glutathione. Trypanothione redox balance is regulated by two enzymes, trypanothione synthase and trypanothione reductase, which are absent in the host and have been chemically and genetically validated as druggable targets [191,192]. Numerous efforts made to synthesize new compounds to target trypanothione reductase in trypanosomatids have delivered novel chemical scaffolds capable of inhibiting this enzyme [193]. Unfortunately, none of these compounds have shown good profiles as drug candidates for further development [194]. DNA topoisomerase IB, the enzyme involved in the relaxation of nuclear DNA from trypanosomatids, differs from the host’s counterpart in structure and gene expression [195]. Genetic disruption of any of the two subunits of DNA topoisomerase IB results in a nonviable phenotype that proves potential draggability of this essential enzyme [196]. Camptothecin-like compounds irreversibly target this enzyme and have shown antileishmanial effects in vivo [197].

Since the most of the lead compounds selected through target-based screening frequently lack activity against the whole cell or in whole organism assays, improved higher performance phenotype-based screens have been introduced in recent decades [174,198]. The goals to be addressed in a phenotypic screening for trypanosomatids are: (i) to find the drug that selectively kills the parasite at the lowest concentration; (ii) to kill the most relevant parasitic form, which is responsible for the pathological outcome in the host, and (iii) to find the safest concentrations for the host’s cells [168,199].

To achieve the first goal, the introduction of bioimaging techniques has accelerated the discovery of new potential hits based on phenotypic screening [199,200]. The use of genetically modified pathogens that express reporter genes encoding colored, fluorescent or luminescent proteins, along with high content screening (HCS) readouts, have facilitated the early discovery of drugs under the phenotypic screening paradigm [201,202]. Genes encoding potential reporter proteins were initially identified in marine organisms and could be easily cloned in suitable vectors for steady expression in the pathogens, preferably using genomic integration strategies [200,202]. The choice of these reporters will depend on the preferable or adequate readout, although some pros and cons should be considered [200]. Color-based readouts lack the sensitivity of those using fluorescence or luminescence ones. In addition, they are only useful for free-living forms of the parasites, unless HCS systems are available to avoid misinterpretation of results [203,204]. Vital dyes like MTT or Alamar Blue, or transgenic parasites expressing intracellular reporter genes like *lacZ* (encoding beta-galactosidase) have been used to test *T. brucei* bloodstream parasites, *T. cruzi* trypomastigotes and axenic *Leishmania* amastigotes [205]. However, these techniques are currently outdated. In order to improve sensitivity and detection of parasites in cocultures with mammalian cells, genetically modified parasites expressing fluorescent proteins are preferred. The advantages of fluorescence emission over colorimetric readings include increased sensitivity, no dye requirement and the possibility of scaling up to certain preclinical models using charge-coupled devices (CCD) with the reporters [206,207]. Genes encoding fluorescent proteins, which emit in the range of UV to near-infrared spectra, are extensively used to create transgenic strains of kinetoplastids [174,200]. Either episomal or integrative transgenic strains of *T. brucei*, *T. cruzi* and several species of *Leishmania* have been used in phenotypic HTS drug discovery campaigns. The use of stable-transfected (genome integrated) pathogen strains over episomally-transfected is preferred because selection drug-pressure is not (always) necessary to avoid the loss of the plasmid vector [208]. Gene integration into the 18s ribosomal (SSU) loci of trypanosomatids warrants genomic stability and does not affect the viability and virulence of the pathogen [209]. Transfected strains with luciferase-encoding genes from either firefly or marine animals are widely used in drug discovery campaigns due to their greater sensitivity and poor autofluorescence-noise. However, specific luciferase substrates, i.e., luciferin or coelenterazine, significantly increase the cost of these assays. [210,211]. Despite these drawbacks, the radiance emitted by the luciferase-transfected strains in the presence of the dye, and the poor autofluorescence background of the tissues, which do not have to be light-excited for visualization, make it possible to acquire in vivo images in real time of internal infections using CCD cameras [212,213,214].

The second goal of phenotypic screening recommends the use of the most pathogenically relevant form of the parasite. In the case of HAT, *T. brucei* bloodstream forms are easy to grow under in vitro conditions which may closely resemble the blood environment where the parasite lives [215]. However, this is not easy to reproduce with other trypanosomatids. *T. cruzi* has an early epimastigote form in the bloodstream that rapidly invades different host cells and transforms into intracellular amastigotes [216]. In the case of *Leishmania* spp., the most relevant pathological form, the amastigote, lives inside the phagolysosomes of the host macrophages. For many years, cell-based screens on free-living forms (promastigotes and trypomastigotes) or axenic amastigotes (a nonnatural extracellular form created under laboratory conditions) were unable to detect active compounds and identified a large number of false-positives [217]. A step forward in the chain of translatability was represented by the phenotypic platforms based on cultures of mammalian cells infected in vitro with the pathogen. Immortalized cell lines like Vero, 3T3 fibroblasts or LS cells, as well as primary cardiomyocytes, were used to evaluate the intracellular development of *T. cruzi* amastigotes [205,216]. Similarly, murine (J744.2 or RAW-transformed monocytes) or human macrophages (THP-1 transformed monocytes) are suitable models to study the intracellular form of *Leishmania* spp [218,219]. However, despite these intracellular cocultures being closer to real-life experimental models and doing away with the need to use of artificial cell stages like axenic amastigotes, some technical problems remain. They include the lower throughput of the platform, the tumoral origin of most of host the cell lines, which can interfere with the results, and the artificial method of infection, including washing steps, which are far from natural conditions.

An alternative method that works in *Leishmania* is the use of primary cultures of splenic explants obtained from infected rodents ex vivo [220]. This method was first used to screen a library of 10,000 compounds using hamster splenocytes infected with *L. infantum* by Melby’s group (University of Texas, USA) [221]. It was subsequently used with lymph node cells of mice infected with a strain of *L. major* that had been previously transformed with the luciferase-encoding gene [222]. This method was later adapted to a murine model of chronic visceral leishmaniasis using a near-infrared fluorescent *L. infantum* strain [206]. A remarkable advantage of using naturally infected host cells is that it can avoid some handling problems, such as the washing and removal steps of noninternalized parasites that often disturb the readings of experimental infections in vitro. In addition, ex vivo splenic explants are 3D-primary cocultures that include other components of the host immune system which may contribute to the clearance of parasite burden [223]. The translatability of this coculture is currently under evaluation. This model may improve the chain of translatability and thus accelerate the drug discovery process for leishmaniasis.

DNDis, MMV and the Tb Alliance in collaboration with the Bill and Melinda Gates Foundation recommend a number of general and specific hit-to-lead criteria for screening against NTDs, malaria and tuberculosis. An initial screen of potency at a single concentration of 10 μM has been recommended, which is considered as an inclusive criterion. The compounds with the best inhibition rates (70–100%) may be screened in a second round to determine their IC_50_ value. The cytotoxicity of the lead compounds is assessed in mammalian cell lines to determine Selective Indexes (SI). The compounds with SI>10 are recommended for early preclinical evaluation using in vivo models of the disease. Complementary in vitro and in vivo assays, including in vivo exposure after oral administration, in vivo efficacy resulting in >70% pathogen reduction, early safety assessment including in vitro cardiotoxicity, AMES test for genotoxicity and tolerability studies are also necessary to describe a putative lead compound. Several rounds of lead expansion and optimization are required to find a promising drug candidate for advanced preclinical and clinical evaluation [220,224,225].

### 5.2. Phenotypic vs. Target-Based Screening in Malaria

Similar to most of the new drug discovery programs, new antimalarial drug discovery platforms have also relied on whole cell pathogen culture-based phenotypic screenings, as well as on molecular target-based HTS approaches [226]. It is remarkable that most of the antimalarial lead compounds identified through parasite culture-based phenotypic screening have shown better rates of success compared to target-based screening regarding their further advancement to lead-optimization and preclinical development pathways [227,228]. However, target-based screening models are still relevant for structure-activity analyses and for the optimization of new antimalarial drug leads [229,230]. The utility of target-based antimalarial screens has been further enhanced by engineering the target action to functional phenotypic cell-based models [231]. For instance, the electron transport chain [232], the protein kinases PfCLK3 [233] and Pfnek-1 [234], the β5 sites of Pf20S proteasome [235] and the mitochondrial enzyme, dihydroorotate dehydrogenase [236,237], have been identified as potential targets for the discovery of novel antimalarial inhibitors. Remarkably, the dihydroorotate dehydrogenase inhibitor, DSM265 [238], has recently demonstrated its efficacy in patients with *P. falciparum* and *P. vivax* malaria infections.

The main strategy for the discovery of antimalarial drugs under the phenotypic-based paradigm is related to the creation of a drug which prevents development and proliferation during the life cycle of malaria parasites. However, the complexity of multiple cellular, physiological and molecular parasite stages represents a challenge in optimizing the therapeutic development of a new drug. The majority of the antimalarial drug discovery and screening programs were developed to cure malaria by acting against the intraerythrocytic asexual blood stages of the parasite [239], which are primarily responsible for severe pathogenesis of the disease and deaths due to malaria infections [240]. However, platforms designed against either sexual (gametocytes) or hepatic stages (hypnozoites) to prevent or to kill dormant stages of malarial infections are already under development. Several HTS methods have been employed to screen compound libraries against blood stages of the malaria parasite [241]. These methods include DNA-binding fluorescent dyes [242], the parasite lactate dehydrogenase assay methods [243] or the use of transgenic parasites expressing luciferase-reporter cassettes [244,245,246,247]. The SYBR^®^ green fluorescence-based screening with blood stage *P. falciparum* cultures has been the hallmark of antimalarial drug discovery for more than a decade [248]. SYBR^®^ green-based assays have been further optimized for antimalarial screening under low levels of parasitemia against clinical field isolates of *P. falciparum* for surveys of drug resistance [249,250] in infections with mixed strains and for the evaluation of antimalarial drug combinations [251]. The flow-cytometric adaptation of SYBR^®^ green assays can determine the efficacy of drugs against specific life-cycle stages of the malaria parasite [252], and also during in vivo screening in a *P. berghei* mouse model of malaria [253,254]. SYBR^®^ Green assay is a faster, less expensive and more reproducible approach than other traditional techniques, and the most widely used in vitro screening approach with the aim of achieving new adaptable and optimized versions [255].

Transgenic *P. falciparum* cell lines with stable high-level firefly luciferase expression have been employed for high throughput antimalarial screening [256,257]. The stable luciferase-expressing cell lines of *P. berghei* have also been employed for noninvasive whole mouse imaging and in vivo antimalarial screening [247,257]. A simple one-step technique based on RNA dye growth inhibition and high-content imaging assay has been developed for antimalarial HTS [258]. The high content live cell imaging platform with an RNA sensitive dye and imaging at timed intervals has been employed to screen a library of marine extracts against *P. falciparum* [259].

Antimalarial drug discovery programs have been further expanded to screen the compound libraries against sexual gametocytes, [260,261]. The sexual gametocytes are responsible for the transmission of malaria [262], while malaria infection in the mammalian host is established initially in the hepatocytes [263]. Several screenings with gametocytes and different throughputs have been developed. They include mainly colorimetric methods [264,265] and genetically modified parasites with a fluorescent/bioluminescent protein labeling and high-content imaging [262,266,267,268,269,270,271]. Robust HTS campaigns have identified several new gametocidal antimalarial drug leads with utility in the prevention of malaria transmission [272,273,274,275,276]. Recently, the efficacy of this technique has been useful in the identification of active compounds against gametocytes that had not been identified in asexual blood-stage assays [272]. In addition to asexual blood-stage parasites and sexual gametocytes, HTS based on liver-stage screens have been developed for antimalarial drug discovery [277,278,279]. These screening models, which identify the antimalarial drugs that are active against dormant *P. vivax* and *P. ovale* hyponozites, have utility for the radical cure and prevention of malaria relapse [280]. A few HTS assay models have been reported for the screening of compound libraries against *Plasmodium* liver stages [279,280,281,282,283]. Remarkably, a recently study published by Antonova-Koch and coworkers, who performed an HTS with more than 500,000 compounds against malaria liver stages using luciferase-expressing *P. berghei* [284], revealed 58 mitochondrial inhibitors and further chemotypes, although no mechanism of action was identified.

## 6. Marine Based Compounds for NTDs and Malaria

Marine-derived compounds already on the market include drugs to treat cancer, viruses, neuropathic pain and even hypertriglyceridemia, but none for the treatment of NTDs or malaria. However, many natural marine products have been reported to show antiprotozoal activity. In addition, marine organisms are a complementary source of chemical entities that can serve as inspiration for the synthesis of novel drugs in the treatment of tropical diseases [285]. The following paragraphs include some relevant examples of secondary metabolites of marine origin that are currently being evaluated as potential drugs for the treatment of NTDs and malaria [286]. 

### 6.1. Algae-Derived Compounds

**Figure 6:**
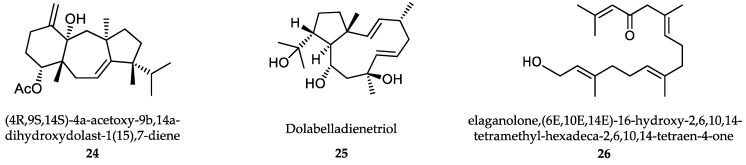
Active metabolites isolated from marine macroalgae.

Benthic marine algae include red (Phyllum Rhodophyta), brown (Phyllum Heterokontophyta, Class Phaeophyceae) and green (Phyllum Chlorophyta) algae. Numerous extracts from marine algae have been evaluated for their antiprotozoal effect. However, only the compounds isolated and identified from these extracts are taken into account in the current review. These compounds include diterpenes, halogenated triterpenes, sulfated polysaccharides, acetogenins, polyphenols and others (Figure 6). A recent review estimated that 151 extracts from up to 30,000 macroalgae species identified worldwide have proven antileishmanial activity [287]. From these, 48 extracts were obtained from brown Phaeophyceae macroalgae, 80 from Rhodophyceae and 23 from green Chlorophytes. Only 12 of these species where further studied to identify bioactive antileishmanial compounds. More than 50% of antileishmanial compounds were major secondary metabolites from brown Dictyotaceae seaweeds with diterpene structure. Due to the relevance of the intracellular amastigote form as the most suitable model for drug-screening, only the results obtained using this parasite stage are presented. The most active of these compounds was the diterpene (4R,9S,14S)-4α-acetoxy-9β,14α-dihydroxydolast-1(15),7-diene, an electron chain transport uncoupler **(24)**, isolated from *Canistrocarpus cervicornis*.

**Figure 7:**
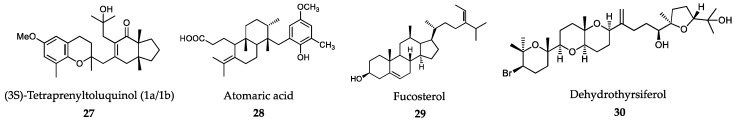
Active metabolites isolated from marine macroalgae.

The IC_50_ value for *L. amazonensis* intracellular amastigotes was 4.0 μg/mL, with an interesting SI of 93 in mouse J774 macrophages [288]. Dolabelladienetriol **(25)** is a diterpene isolated from *Dictyota pfaffii* that inhibited the growth of *L. amazonensis* intracellular amastigotes with a modest IC_50_ value of 43.9 µM and SI>2 on murine macrophages. Dolabelladienetriol modulates macrophage activity by inhibiting NO, TGF-β and TNF-α production, which may explain its antileishmanial activity [289,290]. Bioassay-guided fractionation of extracts from the brown alga *Bifurcaria bifurcata* revealed diterpene elaganolone **(26)** (6E,10E,14E)-16-hydroxy-2,6,10,14-tetramethyl-hexadeca-2,6,10,14-tetraen-4-one, with mild trypanocidal activity against *T. brucei rhodesiense* (IC_50_ = 45 μM and SI 4.0) [291]. Meroditerpenoids, such as (3S)-tetraprenyltoluquinol (1a/1b) **(27)** isolated from extracts of the brown alga *Cystoseira baccata* inhibited the growth of *L. infantum* intracellular amastigotes with an IC_50_ value of 25.0 μM and a SI of 5 on murine macrophages. Mechanistic experiments showed that this compound induced cytoplasmic vacuolization and the presence of coiled multilamellar structures in mitochondria, which produced intense disruption of the mitochondrial membrane potential [292]. Atomaric acid **(28)** is another meroditerpenoid, isolated from extracts of the Caribbean-sea alga *Stypopodium zonale.* It showed an IC_50_ value of 20.2 μM against intracellular amastigotes of *L. amazonensis* and SI value of >8.4. The generation of free radicals may be partially responsible for its antiprotozoal activity [293]. The triterpene fucosterol **(29)** (Figure 7), isolated from the brown macroalga *Lessonia vadosa*, was active against intracellular amastigotes of both *L. amazonensis* and *L. infantum* (IC_50_ values of 7.89 μM and 10.30 μM, respectively) and exhibited relatively low cytotoxicity (CC_50_ >100 μM) [294]. 

**Figure 8:**
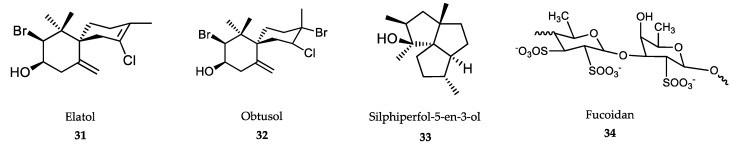
Active metabolites isolated from marine macroalgae.

The weak effect exerted by this compound on promastigotes may indicate that the antileishmanial activity of fucosterol is somewhat dependent on macrophage function [294]. Recently, a large number of triterpene polyether compounds with significant structural and pharmacological diversity were identified in the red alga *Laurencia viridis*. Dehydrothyrsiferol **(30)**, a natural oxasqualenoid, has similar trypanocidal (*T. cruzi*) activity to the reference drug benznidazole **(6)**. However, the SI value was not as safe as that of benznidazole (SI = 3 vs. 56), the clinically used drug. In addition, dehydrothyrsiferol showed an IC_50_ of 2.16 μM against *L. amazonensis* amastigotes, but again, the SI was much lower than that shown by the reference drug miltefosine **(11)**. However, the semisynthetic derivative resulting from the introduction of an iodine atom in these series led to the identification of 28-iodosaiyacenols A and B, which exhibited notable antileishmanial activity and turned out to be nontoxic against the J774 line of murine macrophages [295]. Sesquiterpene derivatives elatol **(31)**, obtusol **(32)** and the triquinane derivative silphiperfol-5-en-3-ol **(33)** (Figure 8), isolated from the red macroalga *Laurencia dendroidea*, were tested against *L. amazonensis*. Unlike silphiperfol-5-en-3-ol (IC_50_ = 48.7 μg/mL), elatol and obtusol were strongly active against intracellular amastigotes (IC_50_ = 4.5 μg/mL and 3.9 μg/mL, respectively). None of these compounds significantly activated NO synthesis by infected macrophages, which suggests that their antileishmanial activity is likely to be exerted on the parasites, rather than through macrophage activation [296,297]. 

The antileishmanial activity of sulfated polysaccharide fucoidan **(34)**, isolated from the brown alga *Laminaria japonica* (Figure 8), was tested in both in vitro and in vivo models of *L. donovani*. Fucoidan was able to kill more than 90% *L. donovani* intracellular amastigotes at 50.6 μg/mL. The effectiveness of this compound for the clearance of parasite burden in liver and spleen was complete in in vivo models of antimony-susceptible and -resistant strains of *L. donovani*. Fucoidan induced a strong Th-1 response in the host by increasing the production of NO, cytokines and free radicals in infected macrophages. Unfortunately, at a dose of 200 mg/kg body weight/day three times daily, this compound showed high hemorrhagic risk and poor bioavailability [298].

### 6.2. Sponge-Derived Compounds 

Sponges produce many different kinds of chemical substances which are active against several pathogens, including virus, bacteria and protozoa [299,300]. However, despite the considerable number of new bioactive compounds that have constantly been isolated from sponges, only a small number has reached the market. 

**Figure 9:**
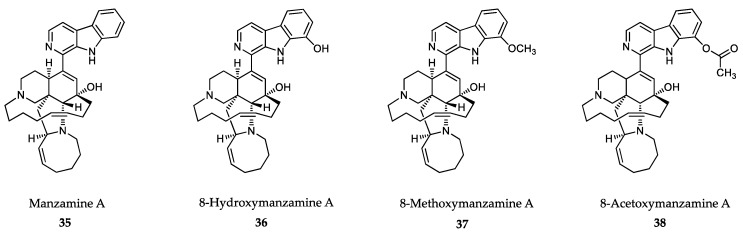
Active metabolites isolated from sponges.

The most promising antiprotozoal compounds isolated from sponges are manzamines (Figure 9). Manzamines are eight-membered β-carboline alkaloids that have served as structural core for a large number of biologically relevant semisynthetic compounds. The isolation, characterization and anticancer effect of manzamine A **(35)** from sponges of the *Haliclona* genus was reported by Sakai and coworkers in 1986 [301]. The first antimalarial effects of manzamine A and 8-hydroxymanzamine A **(36)** against asexual erythrocytic stages of *P. berghei* were reported by Ang and coworkers in 2000 [302]. These authors showed that a single intraperitoneal injection of 100 μmol/kg body weight of manzamine A and 8-hydroxymanzamine-A to *P. berghei*-infected mice prolonged survival time for more than 60 days. In addition, oral administration of manzamine A at a similar dose also produced significant reductions in parasitemia, which indicates good pharmacokinetic properties of these alkaloids [303]. Natural manzamine derivatives showed moderate antileishmanial activity against *L. donovani* promastigotes. However, the semisynthetic derivatives 8-methoxymanzamine A **(37)** and 8-acetoxymanzamine A **(38)** are prodrugs that showed improved antileishmanial and antimalarial potencies with low cytotoxicity [304].

**Figure 10:**
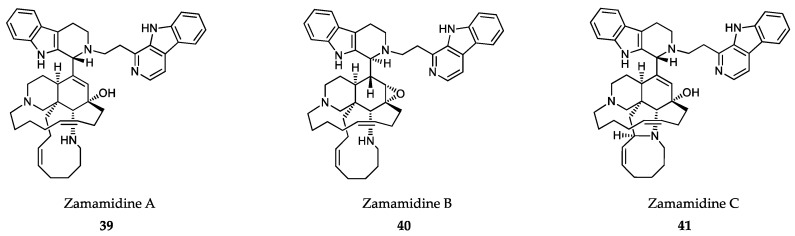
Active metabolites isolated from sponges.

Many other β-carboline alkaloids have been synthesized since then, but they have hardly increased the antiprotozoal potency of the parent compounds [305,306]. Recently, zamamidines A-C **(39-41)** (Figure 10), other manzamine alkaloids isolated from *Amphimedon* sp. sponges, have shown inhibitory activities against *T. brucei brucei* (IC_50_ values ranging from 0.27 mg/mL to 1.05 mg/mL) and *P. falciparum* (IC_50_ values ranging from 0.58 mg/mL to 12.20 mg/mL), with the C form **(41)** being the most active compound of the series [307]. The mechanism of action of manzamine alkaloids is not fully understood, but some authors describe β-carboline alkaloids as micromolar inhibitors of glycogen synthase 3 (GSK-3) from malaria parasites [308] and DNA topoisomerase inhibitors through intercalating into DNA base pairs [309].

**Figure 11:**
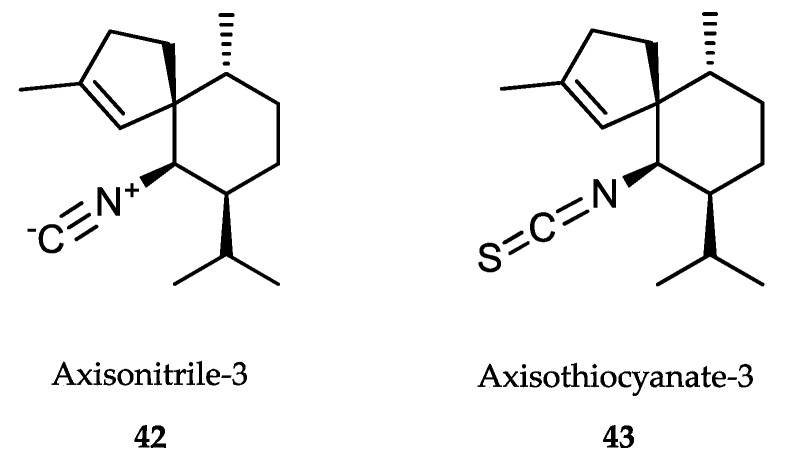
Active metabolites isolated from sponges.

Isonitrile-, isothiocyanate- and formamide-containing sesquiterpenoid metabolites were first isolated from extracts of the sponge *Axinella cannabina* (Figure 11). Axisonitrile-3 **(42)**, unlike axisothiocyanate-3 **(43)**, was found to possess a potent antimalarial activity both on chloroquine-sensitive and chloroquine-resistant *P. falciparum* strains in the nanomolar range. In addition, it showed poor cytotoxicity, which points to the relevance of the isonitrile group [310]. A chemical analysis of the sponge *Cymbastela hooperi* (Axinellidae) provided other diterpene isonitriles, which showed even higher antimalarial effect and moderate toxicity [311]. Docking studies of these compounds with human hemoglobin indicated that the inhibitors directly interacted with the heme group, thus forming a coordination complex with the iron center and inhibiting the transformation of the heme group into β-hematin and then hemozoin [312]. 

**Figure 12:**
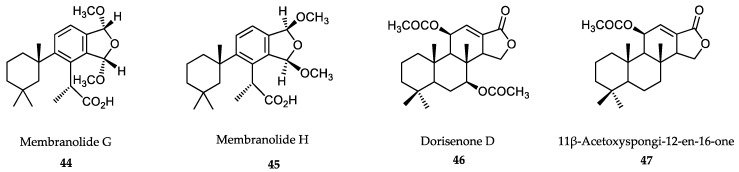
Active metabolites isolated from sponges.

The diterpenes membranolides G **(44)** and H **(45)** (Figure 12), isolated from the sponge *Dendrilla membranosa*, also displayed antileishmanial activity against *L. donovani* axenic amastigotes with IC_50_ values of 0.8 and 1.1. μM, respectively. The latter examples attest to the fact that marine biota can provide novel compounds with antiprotozoal activities if properly assayed [313]. The extracts of marine sponges from the genera *Spongia* and *Ircinia* contain broad-spectrum antiprotozoal meroterpenes, linear triterpenoid and squalene, with good inhibitory effects on major trypanosomatids and *P. falciparum*. The meroterpene dorisenone D **(46)** could become a promising antiplasmodial lead compound. In addition, the diterpene 11β-acetoxyspongi-12-en-16-one **(47)** exhibited strong antileishmanial potential against *L. donovani*, but with low SI [314] (Figure 12).

**Figure 13:**
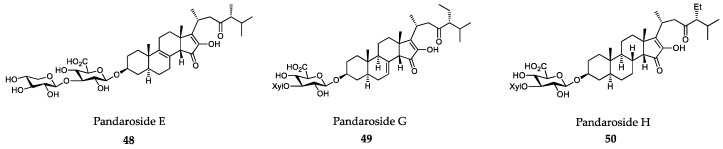
Active metabolites isolated from sponges.

The steroidal glycosides pandaroside E **(48)**, G **(49)** and H **(50)** and their methyl esters isolated from extracts of the Caribbean sponge *Pandaros acanthifolium* strongly inhibited the growth of *T. brucei rhodesiense* (bloodstream forms), *T. cruzi* (intracellular amastigotes in L6 rat skeletal myoblasts), *L. donovani* (axenic amastigotes) and *P. falciparum* at low and submicromolar concentrations (Figure 13) [315].

**Figure 14:**
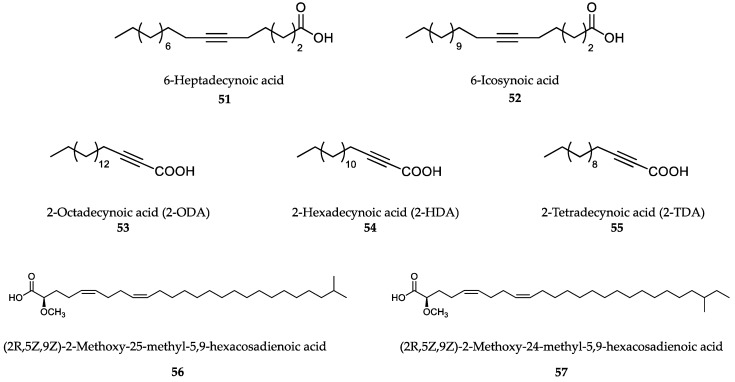
Natural (51,52,56,57) and synthetic (53-55) long-chain unsaturated fatty acids.

The antileishmanial and antimalarial activities of naturally occurring long-chain fatty acids have been recognized for many years [316]. However, an important observation made by Carballeira’s group in both *P. falciparum* and *Leishmania* spp. was that fatty acids should contain a high degree of unsaturation to display antiprotozoal activity (Figure 14) [317]. The naturally occurring acetylenic fatty acids 6-heptadecynoic **(51)** and 6-icosynoic **(52)** acids displayed good antileishmanial activity, with 6-icosynoic acid being the most effective in the series.

The saturated fatty acids n-heptadecanoic acid and n-eicosanoic acid were not effective towards *L. donovani*, which indicates that double or triple bonds are necessary for activity. Interestingly, 6-icosynoic acid **(52)** reversibly inhibited the leishmania DNA topoisomerase IB enzyme (LTopIB), with an IC_50_ between 36-49 μM. [318]. LTopIB was described as a potential target in Trypanosomatids due to its anomalous heterodimeric structure and differential expression in the parasite [319]. Therefore, other unsaturated fatty acids were synthesized to target it [320,321,322]. Within this series, the 2-octadecynoic acid (2-ODA) **(53)**, 2-hexadecynoic acid (2-HDA) **(54)** and 2-tetradecynoic acid (2-TDA) **(55)** were studied on the three major Trypanosomatids. The best effect was achieved on *L. donovani* amastigotes (IC_50_ ranging from 11.0 to 24.7 μM), whereas the worst results were obtained against *T. cruzi* (IC_50_ between 62.4 and 80.0 μM) and *T. brucei rhodesiense* (IC_50_ between 64.5 and 255.4 μM) (trend: 2-ODA > 2HDA > 2TDA) [323]. More recently, a series of α-methoxylated Δ5,9 fatty acids **(56,57)** from the sponges *Asteropus niger* and *Erylus goffrilleri* showed antileishmanial activity against intracellular amastigotes of *L. infantum.* These compounds inhibited LTopIB by means of a mechanism different from that of camptothecin, the reference inhibitor of TopIB enzymes [324,325]. The series of C16 isomeric acetylenic fatty acids was tested in vitro against the blood stage of *P. falciparum* and the liver stage of *P. yoelii*, and 2-HDA was found to be the most effective fatty acid (IC_50_ = 41.2 μM) with no cytotoxic effects on mammalian cells [326]. 

The 2-alkynoic fatty acids inhibit the so-called type II fatty acid synthase (FASII), an exclusive eight-enzyme complex involved in fatty acids synthesis in plasmodium parasites. Also, 2-ODA **(53)** and 2-HDA **(54)** showed good inhibition of three enzymes of the complex (IC_50_ 1-2 μM) by binding to a different site from that of the substrate or the cofactor [327].

### 6.3. Metabolites Derived from Other Invertebrates

**Figure 15:**
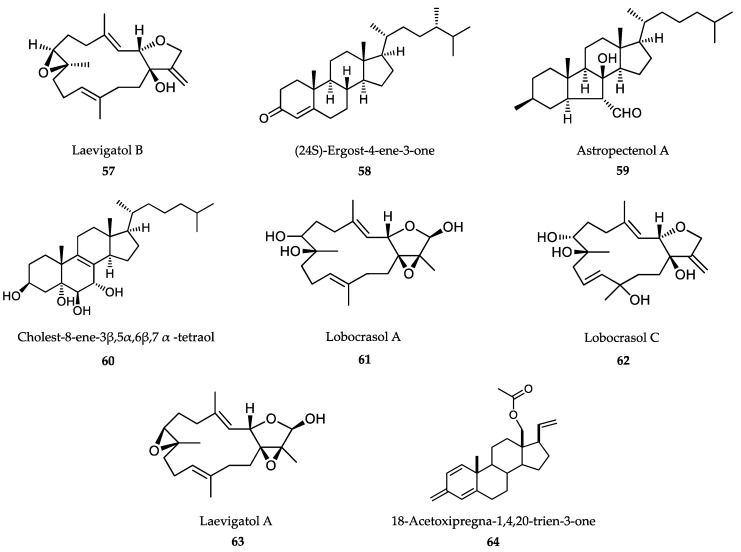
Active metabolites isolated from marine invertebrates other than sponges.

Cnidarians produce or accumulate a diverse array of biologically active secondary metabolites including steroids, terpenes, acetogenins, alkaloids and polyphenolics (Figure 15). They are excellent candidates for development as pharmacological probes, especially for the treatment of NTDs [328]. To identify inhibitors of *T. brucei* growth, a phenotypic HTS based on resazurin staining was carried out with a library consisting of 861 purified natural products and subfractionated extracts (including 433 extracts and 428 pure compounds) from soft corals and echinoderms living in Vietnamese seas (genus *Lobophytum*, *Sinularia*, *Astropecten* and *Diadema*). Several compounds isolated from these extracts, like laevigatol B **(57)**, (24S)-ergost-4-ene-3-one **(58)**, astropectenol A **(59)** and cholest-8-ene-3β,5α,6β,7α-tetraol **(60)**, showed *T. brucei* antrypanosomal activity and also exhibited significant inhibitory effects on *T. cruzi*. By using a high content analysis Operetta device, all these compounds showed anti-inflammatory or anticancer properties in previous studies [329]. Interestingly, in order to increase the antiprotozoal spectrum of these compounds, 34 of the active cembranoid diterpenes selected from previous work were tested against *L. donovani* and *P. falciparum*. From these compounds, lobocrasol A **(61)** and lobocrasol C **(62)** were strongly and selectively active against *L. donovani*. Only one compound, laevigatol A **(63)**, displayed antiplasmodial activity with an IC_50_ < 5.0 μM, and can therefore be considered moderately active. None of the compounds displayed significant cytotoxicity [330].

The steroid 18-acetoxipregna-1,4,20-trien-3-one **(64)**, isolated from the cnidarian *Carijoa riisei*, was assayed against *L. chagasi* and *T. cruzi* parasites. While the trypomastigotes of *T. cruzi* were susceptible, with a moderate IC_50_ value of 50.5 μg/mL, *L. chagasi* promastigotes were much more susceptible, with an IC_50_ value of 5.5 μg/mL. Nevertheless, the usefulness of this compound is compromised due to its high cytotoxicity on macrophage cells [331].

## 7. Conclusions and Future Perspectives

NTDs represent one of the biggest challenges for a changing humanity, in which urban concentration and global warming favor the transmission of these diseases and threaten the most disadvantaged sectors of the population. Furthermore, migratory fluxes increase the risks in regions of the planet that are currently not endemic. In addition to these problems, there is a lack of prophylactic treatments in the form of effective vaccines, which means that prevention falls to public health policies, the latter being often ineffective in countries threatened by more urgent problems. However, it is clear that pharmacological treatment is the only effective solution after the onset of symptoms. Throughout this review, it has become evident that all existing treatments for NTDs in the 21st century are over 40 years old. Although they have saved hundreds of thousands of lives, many have lost their initial efficacy and generated resistance in certain regions where they are currently not effective. In a globalized world, the existence of these diseases has become ethically intolerable, and a multitude of initiatives have emerged for their total eradication. Supranational entities such as the DNDi, the Bill and Melinda Gates Foundation (Seattle, WA, USA), private companies, NGOs and public administrations, among others, signed the London Declaration on NTDs on 30 January 2012 in London. This initiative launched a collaborative disease eradication program inspired by the WHO 2020 road map. Since then, the efforts made have been remarkable, but only a handful of alternative treatments, many of them consisting of a combination of existing drugs or optimized treatments for certain sectors of the population, have improved the previous situation. However, new drugs that are more effective than existing ones have not emerged. It should be emphasized that existing treatments, in addition to causing resistance, must be administered parenterally. This reduces adherence to prolonged treatments and, in many cases, involves patient hospitalization. In addition, and more importantly, they are also responsible for significant adverse reactions. 

For this reason, the screening of the current chemical space in optimized in vitro systems has become the most promising option for these diseases. This strategy, which has been successfully applied to other important diseases, such as cancer, cardiovascular and neurological diseases, amongst others, comes late for NTDs. In order to find new drug candidates that fill the gap in preclinical models of the disease, the development of accurate in vitro models and access to the libraries of hundreds of thousands of compounds of large companies and institutions are necessary. In addition, the introduction of omics techniques has shed light into parasite biology and their interaction with the host. An example is the limitation of long-term culture of *P. vivax*, where single-cell omics is bringing new perspectives to solve complex problems. Metabolomics that lie at the end of the omics cascade has been used to reveal the mechanism of action of some antiparasitic drugs, such as beznidazole against *T. cruzi* or the natural resistance to antileishmania drugs of the clinical isolates of *L. donovani*.

The existence of relevant in vitro models of NTDs for application to HTS platforms is a relatively recent event. The application of bioimaging systems to phenotypic screens has improved the translatability to preclinical and clinical reality, and has served to identify a plethora of new chemical entities with potential antimalarial or antitrypanosomal effect (including *Leishmania*). However, despite the richness and diversity of compounds of marine origin, most of the chemicals from marine environments with pharmacological use for NTDs, including malaria, have been identified using low-performance techniques in in vitro, and sometimes, in in vivo models.

Accessing the chemical diversity of natural products is a major challenge for many modern drug discovery programs. This is largely due to the willingness to examine libraries of pure compounds, which raises problems regarding the availability and supply of compounds. New programs and/or foundations with important natural product repositories have made their libraries of natural products, extracts and facilities available to academic researchers. This circumstance and the collaboration with supranational institutions may help in the identification of new compounds with better pharmacological and toxicological profiles for the treatment of these diseases. From these collaborations, new molecules against NTDs and malaria are expected to emerge in the current decade. They will be added to existing treatments for the eradication of some of the diseases covered in this review.

## Figures and Tables

**Table 1:** Current pharmacological treatments for trypanosomatid-borne diseases and malaria and their doses.

Disease/species	Drug		Chemical Class/Origin	Dose	Reference
*Human African Trypanosomiasis (HAT)*	Pentamidine		Aromatic diamidine	Deep intramuscular injection 4 mg/kg body weight daily for 7 days	[57,58,63]
	Suramin		Polysulphonated naphthylurea	Slow intravenous infusion Day 1: 5 mg/kg body weight Day 3: 10 mg/kg body weight Days 5, 11, 17, 23, 30: 20 mg/kg body weight	[57,64,65]
	Melarsoprol		Organic arsenical	Slow intravenous infusion 2.2 mg/kg daily for 10 days	[57,71]
	Eflornithine		Amino acid analogue	Intravenous infusion 100 mg/kg body weight four times daily for 14 days	[57,79,80,81]
*American trypanosomiasis*	Benznidazole		Nitroheterocyclic compound	Oral 5-7 mg/kg body weight for 60 days	[95,96,97]
	Nifurtimox		Nitroheterocyclic compound	Oral 8–10 mg/kg body weight for 90 days	[103,104,105]
*Leishmaniasis*	Pentavalent antimony compounds		Antimony derivative	Intramuscular injection (Pentostam): 20 mg/kg body weight/day for 30 days	[113,114,116]
	Amphotericin B		Polyene antibiotic	1 mg/kg body weight per day 15 - 20 intravenous infusion of deoxycholate amphotericin Intravenous infusions of liposomal amphotericin: single dose 10 mg/kg body weight	[116,120,123]
	Miltefosine		Alkyl-phospholipid	Patients with 30-44 kg: one 50 mg capsule twice daily for 28 consecutive days Patients with ≥45 kg: one 50 mg capsule three times daily for 28 consecutive days	[126,129,130,131]
	Paromomycin		Aminoglycoside antibiotic	Intramuscular injection 11 mg/kg body weight for 21 days	[133]
*Malaria*	Artemisinin	Artesunate	Sesquiterpene lactone derivative	4 mg/kg body weight daily, with a daily dose range of 2-10 mg/kg body weight.	[15,139,143]
		Artemether	Dihydroartemisinin	3.2 mg/kg body weight by immediate intramuscular injection, followed by 1.6 mg/kg daily	[15,140,143]
	Quinoline derivatives	Mefloquine	Quinoline	25 mg/kg body weight/day for 3 days	[143,149]
		Amodiaquine	4- aminoquinoline	10 mg base body weight daily for 3 days	[143,154]
		Tafenoquine	8-aminoquinoline	Single oral dose of 300 mg	[15,143,156,158]
		Primaquine	8-aminoquinoline	0.25 mg/kg body weight daily for 2 weeks	[15,143,156,159]
	Sulfadoxine/pyrimethamine		Sulfadoxine: a synthetic analog of para-aminobenzoic acid (PABA) Pyrimethamine: a synthetic derivative of ethyl-pyrimidine	25 mg/kg body weight sulfadoxine + 1.25 mg/kg body weight pyrimethamine	[15,19,143,161,162]

## Abbreviations

NTDs	Neglected Tropical Diseases;
WHO	World Health Organization;
HTS	High-Throughput Screening;
FDA	Food and Drug and Administration;
HAT	Human African Trypanosomiasis;
DALY	Disability Adjusted Life Year;
NECT	Nifurtimox-Eflornithine Combination Therapy;
CNS	Central Nervous System;
ODC	Ornithine Decarboxylase;
NTR-1	type 1 nitroreductase;
PKDL	Post-Kala-azar Dermal Leishmaniasis;
AmpB	Amphotericin B;
ACT	Artemisinin-based Combined Therapies;
DNDi	Drugs for Neglected Diseases Initiative;
MMV	Medicine for Malaria Venture;
NGOs	Non-Governmental Organizations;
NCI	National Cancer Institute;
NPNPD	Program for the Discovery of Natural Products;
QSAR	Quantitative Structure-Activity Relationship;
CRK12	Cdc2-related kinase 12;
PTR1	Pteridine Reductase 1;
DHFR-TS	bifunctional Dihydrofolate Reductase-Thymidylate Synthase;
HCS	High Content Screening;
CCD	charge-coupled device;
SI	Selective Index;
LTopIB	*Leishmania* DNA topoisomerase IB.
